# Near-infrared fluorescence tattooing: a new approach for endoscopic marking of tumors in minimally invasive colorectal surgery using a persistent near-infrared marker

**DOI:** 10.1007/s00464-023-10491-2

**Published:** 2023-10-23

**Authors:** Michael Thomaschewski, Michael Lipp, Carsten Engelke, Jonas Harder, Isabell Labod, Tobias Keck, Karin Mittmann

**Affiliations:** 1 Department of Surgery, University Medical Center Schleswig-Holstein, Campus Lübeck, Lübeck, Germany; 2 Department of Surgery, Clinic for Gastrointestinal and Colorectal Surgery, Asklepios Klinik Barmbek, Hamburg, Germany; 3 Medical Clinic I, University Medical Center Schleswig-Holstein, Campus Lübeck, Lübeck, Germany; 4 Department of Gastroenterology, Hepatology & Interventional Endoscopy, Asklepios Klinik Barmbek, Hamburg, Germany; 5 EUREGIO BioMedtech Center, University of Applied Sciences Münster, Stegerwaldstr. 39, 48565 Steinfurt, Germany

**Keywords:** Near-infrared fluorescence, Endoscopic tissue marker, Endoscopic tattoo, Near-infrared marker, Fluorescence-guided minimally invasive surgery

## Abstract

**Introduction:**

Intraoperative accurate localization of tumors in the lower gastrointestinal tract is essential to ensure oncologic radicality. In minimally invasive colon surgery, tactile identification of tumors is challenging due to diminished or absent haptics. In clinical practice, preoperative endoscopic application of a blue dye (ink) to the tumor site has become the standard for marking and identification of tumors in the colon. However, this method has the major limitation that accidental intraperitoneal spillage of the dye can significantly complicate the identification of anatomical structures and surgical planes. In this work, we describe a new approach of NIR fluorescent tattooing using a near-infrared (NIR) fluorescent marker instead of a blue dye (ink) for endoscopic tattooing.

**Methods:**

AFS81x is a newly developed NIR fluorescent marker. In an experimental study with four domestic pigs, the newly developed NIR fluorescent marker (AFS81x) was used for endoscopic tattooing of the colon. 7–12 endoscopic submucosal injections of AFS81x were placed per animal in the colon. On day 0, day 1, and day 10 after endoscopic tattooing with AFS81x, the visualization of the fluorescent markings in the colon was evaluated during laparoscopic surgery by two surgeons and photographically documented.

**Results:**

The detection rate of the NIR fluorescent tattoos at day 0, day 1, and day 10 after endoscopic tattooing was 100%. Recognizability of anatomical structures during laparoscopy was not affected in any of the markings, as the markings were not visible in the white light channel of the laparoscope, but only in the NIR channel or in the overlay of the white light and the NIR channel of the laparoscope. The brightness, the sharpness, and size of the endoscopic tattoos did not change significantly on day 1 and day 10, but remained almost identical compared to day 0.

**Conclusion:**

The new approach of endoscopic NIR fluorescence tattooing using the newly developed NIR fluorescence marker AFS81x enables stable marking of colonic sites over a long period of at least 10 days without compromising the recognizability of anatomical structures and surgical planes in any way.

Colorectal cancer is the third most common cancer type and second main cause of cancer-related deaths in the world with nearly 1.9 million new cases and 916,000 deaths in 2020 [1]. Despite recent developments of new multimodal approaches for colorectal cancer treatment, including targeted immunotherapy or total neoadjuvant therapy, surgical resection remains the primary and most important cancer treatment [2–4]. With the introduction of minimally invasive surgery (MIS), significant advances have been achieved in the surgical treatment of colorectal carcinoma. Several international multicenter randomized trials have shown that laparoscopic resection of localized colorectal cancer results in equivalent oncologic outcomes compared with open surgical resection, but with shorter hospital stay and other perioperative short-term outcome improvements [5–11]. While tumors at the coecum and in the rectum can be reliably localized during preoperative colonoscopy or rectoscopy, reliable localization in the other areas of the colon is uncertain using preoperative diagnostic colonoscopy alone [12, 13]. In contrast, intraoperative tumor localization by endoscopy is logistically demanding, time-consuming, and may induce bowel distension resulting in reduced visibility during MIS [14]. In the case of larger tumors, the location in the colon can usually be determined preoperatively by computed tomography (CT) or magnetic resonance imaging (MRI) [15]. However, CT or MRI does not reliably localize small lesions in the colon. In 1975, endoscopic injection of a blue dye in the submucosal space was firstly described for preoperative marking of small colon tumors. This approach also named as tattooing enables a permanent marking of lesions in the colon and rectum. Since 1975, India ink has largely been used for endoscopic marking of colonic lesions. However, several side effects have been described using India ink for endoscopic tattooing including abscess formation, focal peritonitis, inflammatory tumors, idiopathic inflammatory bowel disease, or adhesion-related ileus [16–20]. These India ink side effects led to the development of a new agent for endoscopic tattooing similar to India ink. The SPOT™ is a suspension based on smaller carbon particles without proinflammatory additives. Its safety and efficacy in terms of staining permanence and visibility have been proven in a large series of patients [21]. Both India ink and SPOT™ allow long-lasting marking of lesions for up to several years [22] making them also ideal for marking of suspicious lesions that require endoscopic follow-up. In MIS colon surgery, endoscopic tattooing is currently considered the method of choice for preoperative marking of small tumors or lesions [13]. During endoscopic tattooing, several submucosal injections are placed circumferentially adjacent to the lesion in order to maximize the surgeon´s ability to localize the lesion intraoperatively in MIS [13]. Nonetheless, incidental intraperitoneal spillage of endoscopically applied tattooing agents was reported in 2.4% to 13% of all cases resulting in a significant reduction in the visibility and identification of the anatomical structures and planes during surgery [14, 23]. Figure 1 shows an example from our own clinical practice: during laparoscopy, the endoscopic tattoo in the ascending colon is clearly visible (Fig. 1). However, due to intraperitoneal spillage of the dye, the surrounding anatomical structures were also intensively stained (Fig. 1). This significantly complicated the identification of anatomical structures and surgical planes. However, in oncologic colon surgery, surgical resection in the embryological planes is highly recommended to improve long-term oncological outcomes, also referred to as the surgical principle of complete mesocolic excision (CME) [24]. Intraperitoneal spillage of India ink or SPOT™ significantly complicates the identification of the embryological planes and thus carries the risk that the surgical oncologic radicality cannot be ensured or even intraoperative complications occur.

**Fig. 1:**
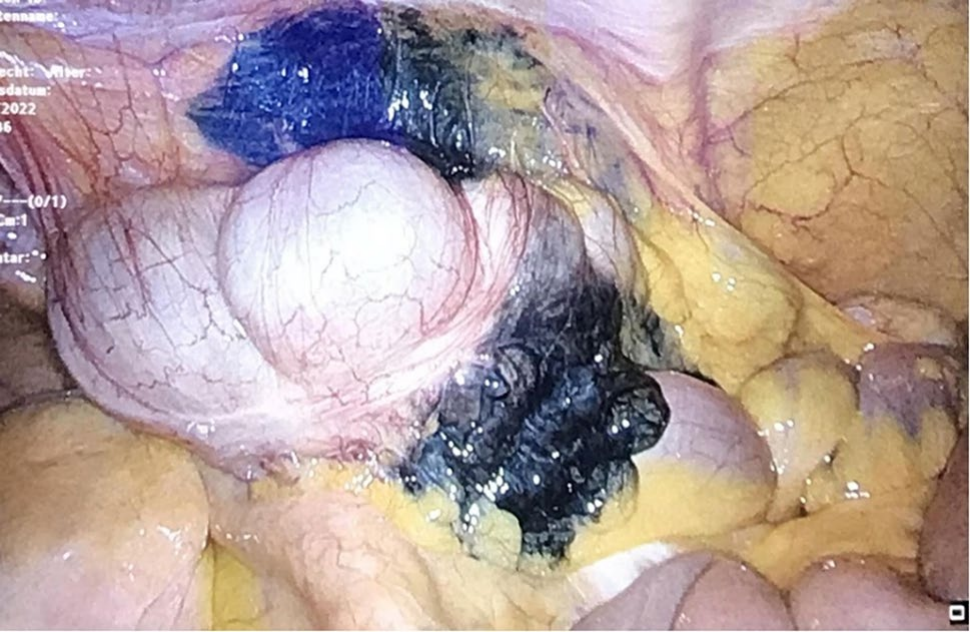
Intraoperative spillage after endoscopic marking (tattooing) of a tumor located in the ascending colon

In addition, colon tumors can be marked preoperatively by endoscopic application of a metal clip. In contrast to endoscopic tattooing, endoscopic clip marking cannot result in a reduced visibility of anatomical structures and planes during surgery. However, intraoperative localization with a C-arm requires the use of X-rays and is logistically demanding and time-consuming [14]. In addition, the clips may spontaneously detach from the mucosa, which then makes intraoperative tumor identification possible only by intraoperative colonoscopy [25]. In consequence, endoscopic clip marking has not been widely used to mark lesions in the colon.

In conclusion, current available methods for preoperative localization of colon tumors have relevant limitation, since they do not meet all the requirements of a reliable and efficient marker for MIS colon surgery as they are (i) high intraoperative visibility of marking without limiting the recognition of the anatomical structures and planes, (ii) high location stability over a long period of time, (iii) high level of patient safety, and (iv) not time-consuming and logistically demanding during surgery.

In this work, we firstly describe a new approach for endoscopic tattooing using the NIR fluorescent marker AFS81x enabling both high intraoperative visibility without limiting the recognition of the anatomical structures and high location stability over a long period of time. This resulted in the development of an entirely new approach of endoscopic marking of colonic lesions, which we have named NIR fluorescence tattooing.

## Materials and methods

The conduct of this study on endoscopic tattooing with a new NIR fluorescent marker was applied for and approved by the Brandenburg State Office for Occupational Safety, Consumer Protection, and Health under application number 2347-06-2022-24-G. With this approval, no additional Institutional Review Board (IRB) approval was required to conduct the study. The animal experimental work was performed at the Medical Competence Center, Wendisch Rietz, Germany, in compliance with applicable animal welfare regulations. Both surgeons evaluating endoscopic markings during laparoscopy signed an informed consent to participate in the study.

### Near-infrared (NIR) fluorescent marker AFS81x

AFS81x is a newly developed NIR fluorescent marker consisting of a sterile polymer microparticle suspension for injection. The microparticles contain fluorescent cadmium-free quantum dots with an emission maximum at 810 nm. The sterile suspension of AFS81x-polymer microparticles was kindly provided by KanMedim UG, Steinfurt, Germany, for this study.

### Endoscopic application of the fluorescent marker

Endoscopic tattooing was performed in four domestic pigs with a body weight between 55 and 62 kg. Using a flexible endoscope (Silver Scope Gastroscope, Karl Storz SE & Co. KG, Germany), marker was placed in the endoscopically accessible part of the colon after bowel cleansing. Depending on the endoscopically individually accessible length of the colon (approx. 25–40 cm), 7–12 markers were placed per animal in a total of four pigs. For this purpose, 0.5 mL each of the fluorescent marker AFS81x was injected into the submucosa with a suitable sclerosing needle (2300 mm length, outer diameter 2.4 mm, 23 G, 6 mm needle length, Keysurgical, Germany) by the gastroenterologists involved in the study on day 0.

### Laparoscopic visualization of the NIR fluorescence signal

On day 0, immediately after endoscopic application of the fluorescent marker, the visualization was evaluated, and photographically documented by the surgeons using a fluorescence laparoscope (Image1 S Rubina with Hopkins optics, Karl Storz SE & Co. KG, Germany).

The diameter of these markings was determined by comparison with a tactile stick with cm markings (surgical instruments, Karl Storz SE & Co. KG, Germany). The settings of the laparoscopic system were identical for day 0, day 1, and day 10 to ensure comparability of the laparoscopic results for the evaluation of the quality of the marking. Laparoscope 0° Hopkins optics, a light intensity of 55%, and overlay mode with an intensity of 75% were used on all examination days.

### Histologic examination of ex vivo tissue

On day 10, the colorectum was resected after the end of the study. The fluorescently labeled injection sites and fluorescent lymph nodes were marked in the specimen by the clinical surgeons using suture material for retrieval during histologic examination. The harvested tissue was then fixed in 5% formalin solution. Fluorescence labeled colon areas (approximately 20 × 4 mm) and labeled lymph nodes were dissected by the Institute of Animal Pathology, Berlin, Germany. After paraffin embedding, sections with 2 µm slice thickness were stained with hematoxylin/eosin and compared with control tissue (non-fluorescent colon wall or lymph nodes).

## Results

**Fig. 2:**
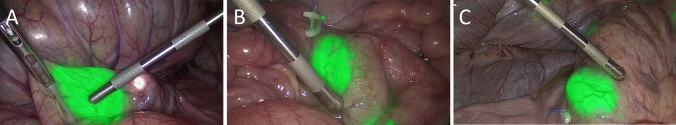
Visualization and identification of the same NIR fluorescent tattoo in the colon during laparoscopy on **A** day 0, **B** day 1, and **C** day 10 after endoscopic tattooing using the fluorescence dye AFS81x. The brightness, the sharpness, and the diameter remained constant even 10 days after endoscopic submucosal injection

A total of *n* = 38 NIR fluorescent tattoos were endoscopically placed in the colon of four pigs by endoscopic submucosal injection of the fluorescent marker AFS81x. During laparoscopy of pigs, visualization and identification of NIR fluorescent tattoos in the colon were performed by an overlay of the white light channel and the NIR channel of the laparoscope (Fig. 2). Thereby, the detection rate of the NIR fluorescent tattoos at day 0, day 1, and day 10 after endoscopy was 100% for both surgeons.

**Fig. 3:**
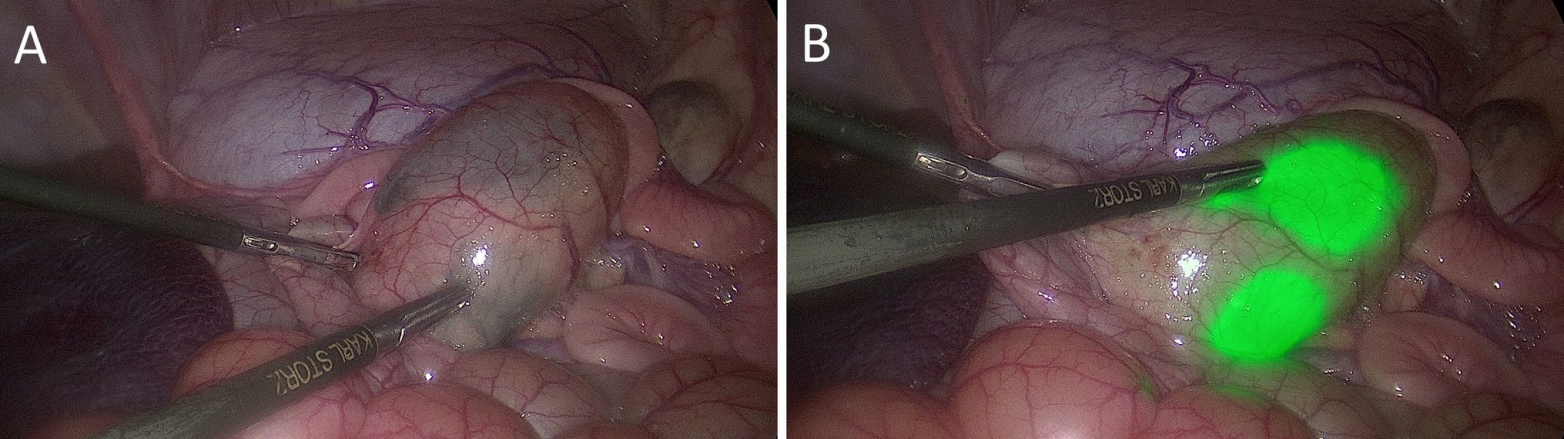
**A** Identification of endoscopic AFS81x fluorescent tattoos and assessment of recognizability of anatomic structures during laparoscopy in white light versus. **B** An overlay of the white light channel and the near-infrared channel of the laparoscope. Since the AFS81x fluorescence marker hardly diffuses into the surrounding tissue after endoscopic submucosal injection, even two closely adjacent endoscopic NIR fluorescent tattoos can be easily distinguished from each other 1 day after the endoscopy (**B**)

The brightness of the endoscopic tattoos was rated by the surgeons using a Likert derived scale (0 to + 3) with + 3 for all of the 38 tattoos on day 0, day 1, and day 10, respectively. The sharpness of the endoscopic tattoos was assessed the same way using the Likert derived scale from 0 to + 3. During in vivo laparoscopy, the sharpness of NIR fluorescent tattoos was scored by the surgeons identical for each tattoo as + 2 on day 0. A slight decrease in sharpness was observed subsequently, leading to an average score of + 1.8 on day 1 and of + 1.9 on day 10. When assessing the recognizability of anatomic structures during laparoscopy after endoscopic NIR fluorescent tattooing, the surgeons were able to recognize 100% of the anatomical structures at all of the 38 marking sites without restriction on all laparoscopic examination days (for example see Fig. 3).

**Fig. 4:**
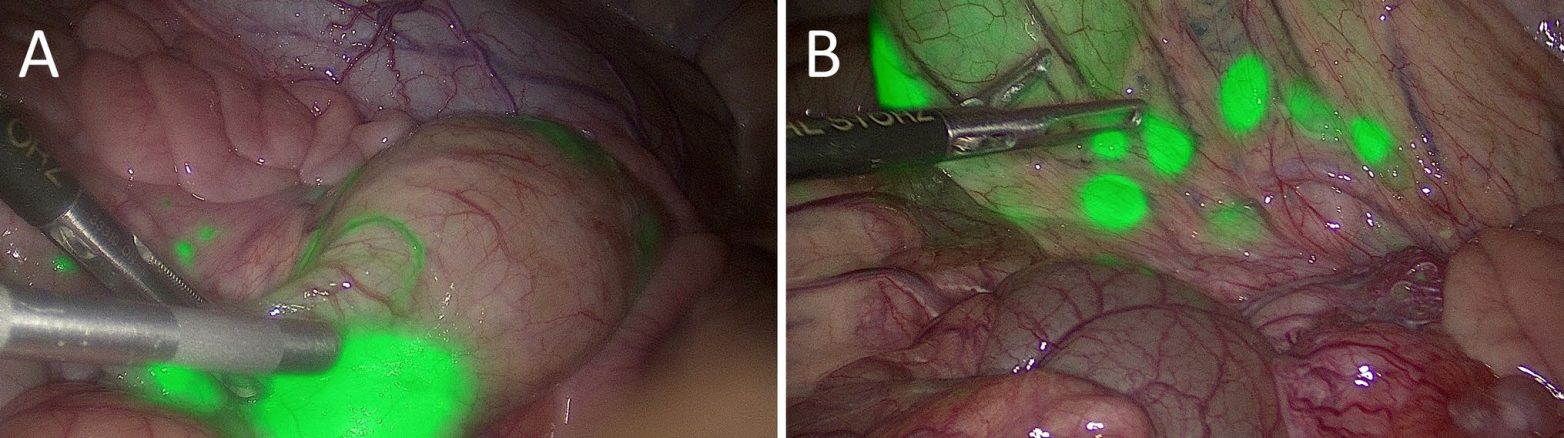
Identification of locoregional lymphatics (**A**) and lymph nodes (**B**) on day 1 after endoscopic submucosal injection of NIR fluorescent dye AFS81x

From day 1 after endoscopic NIR fluorescent tattooing, lymphatics and regional lymph nodes located near the NIR fluorescent tattoos also showed staining with the NIR fluorescent marker (Fig. 4). Histologic examination of the colonic specimens resected on day 10 revealed that macrophages phagocytosed the NIR fluorescent dye AFS81x and transported it by migration to the lymphatics and lymph nodes. In contrast, the NIR fluorescent dye AFS81x was not detectable in the blood serum of the pigs.

**Fig. 5:**
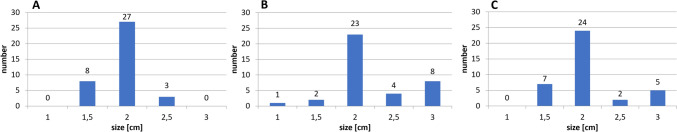
Distribution of endoscopic NIR fluorescence tattoo diameters at **A** day 0, **B** day 1, and **C** day 10 after endoscopic submucosal injection of NIR fluorescent marker AFS81x

The diameter of 38 fluorescence colon tattoos was estimated using a laparoscopic tactile stick with cm scaling. During day 0 laparoscopy, 71% of all endoscopic NIR fluorescent tattoos had a diameter of 2 cm, followed by 21% with a diameter of 1.5 cm and 8% with a diameter of 2.5 cm. During laparoscopy on day 1, most endoscopic NIR fluorescent tattoos remained 2 cm in diameter (61%). However, slightly larger diameter tattoos of 2.5 cm (11%) and 3 cm (21%) were also present. In addition, 3 markings were found whose diameter was smaller than 2 cm. The distribution of the different sizes of endoscopic NIR fluorescent markings at day 10 after endoscopy did not change significantly compared to day 1 (63% with a diameter of 2 cm, 5% with a diameter of 2,5 cm, 13% with a diameter of 3 cm, and 18% with a diameter < 2 cm) (Fig. 5). All animals (*n* = 4) survived in healthy condition and showed no side effects of endoscopic NIR fluorescent tattooing.

## Discussion

In oncologic colon surgery, the precise identification of the location of colonic lesions is necessary to ensure adequate oncologic resection margins and the extent of lymphadenectomy. In MIS, the colonic lesions cannot be adequately identified by palpation since haptic sensation is significantly reduced in MIS or even completely eliminated as in current robotic surgery platforms. In MIS, the surgeon therefore needs visual guidance on the exact localization of tumors in the colon. Especially if the tumors are located in areas near the right or left flexure or their growth does not reach the serosa, visual localization is practically impossible without assistance. Even in open surgery, it can sometimes be challenging to identify small tumors in the colon only by palpation. Endoscopic tattooing of the colonic lesions prior to surgery has become widely established as the method of choice, as India ink and SPOT™ tattoos of the colon can be detected readily and accurately during MIS without the need of additional technical requirements [13]. A major drawback of endoscopic tattooing is that intraoperative identification of anatomic structures and surgical planes can be significantly compromised after an accidental intraperitoneal injection. This can substantially complicate the surgical procedure [14]. And, accidental intraperitoneal spillage of endoscopically applied India ink or SPOT™ is not a rare event, but occurs in 2.4% to 13% of all cases [14, 23].

In this work, we describe for the first time a new approach for endoscopic tattooing of colonic lesions using a new NIR fluorescent marker instead of India ink and SPOT™. We named this new method “NIR Fluorescence tattooing.” Because the NIR fluorescent marker is not visible in white light, there is no risk of limiting the identification of anatomical planes and structures if the marker is accidentally spilled intraperitoneally. We demonstrated that endoscopic tattoos with this new NIR fluorescent marker can be readily and accurately detected during MIS for a period of at least 10 days. In our animal study, we confirmed a 100% detection rate of NIR fluorescent tattoos by two surgeons during MIS even 10 days after endoscopic tattooing. In our study, we also demonstrated that the NIR fluorescent marker did not fade or spread 10 days after submucosal endoscopic injection. Instead, the marking with the new NIR fluorescent marker remained localized, permanently sharply defined, and with consistent brightness in NIR imaging, as assessed by two MIS surgeons. Despite intraperitoneal spillage of the NIR fluorescent marker, which also occurred in our study, the detectability and recognition of anatomical structures and surgical planes were not affected by the NIR fluorescent dye in any way, as it is not visible in white light during MIS. This provides a significant advantage of our new approach of NIR fluorescence tattooing over tattooing with India ink and SPOT™.

Other scientific groups have already attempted to use NIR imaging to mark colon lesions. Most groups used indocyanine green (ICG) for endoscopic tattooing instead of India ink and SPOT™ because ICG is only visible in NIR imaging and not in white light. These approaches are similar to ours, except that we used a different new NIR fluorescent marker that, unlike ICG, does not fade or disappear over time. In a study by Watanabe et al., colonic lesions of 80 patients were marked by submucosal injection with ICG [26]. However, not all endoscopic ICG markings could be identified during surgery in this study, but only 93.8%. Thus, in 5 patients, the ICG tattoos in the colon could not be identified during surgery. Even more, in the study, the visibility rate of ICG tattoos was also significantly poorer when the interval between endoscopy and surgery was ≥ 10 days (*p* < 0.001) [26].

In a further study by Watanabe et al. surgery was performed subsequently to endoscopic marking with ICG without a time delay [27]. Here, the group was able to achieve a 100% detection rate of ICG markings in the colon. In a prospective study, Nagata et al. compared endoscopic tattooing with ICG and India ink [28]. Again, surgical intervention was performed subsequent to endoscopic marking because the group was concerned that the ICG markings would fade and disappear over time, thus becoming undetectable. In their study, Nagata et al. showed that fluorescent ICG tattoos improved tumor detection rates compared with gross macroscopic color perception. These data suggest that NIR fluorescence imaging provides high sensitivity in tumor detection [28]. According to the results of both research groups, endoscopic tattooing with ICG has a major limitation, namely the restricted time interval between endoscopic marking and surgery, which should ideally be less than 7 days to ensure reliable detectability of endoscopic markings. However, in further studies concerning endoscopic ICG tattooing, the ideal time interval between endoscopic marking and surgery was even shorter, namely 1 to 3 days [14]. Moreover, being a small hydrophilic molecule, ICG tends to diffuse widely into the tissue. In fact, ICG fluorescence diffusion has been reported up to 7 cm from the injection site [29], which could theoretically lead to misdetection of colon lesions.

Another approach addressing this limitation of endoscopic ICG tattooing was recently described by Barberio et al. [30]. The research group of Barberio et al. engineered a fluorescently coated over-the-scope clip (OSC), using a biocompatible polymer. Since the over-the-scope clips do not detach even 2 months after their application [31], their fluorescence signal could provide reliable detection of endoscopic markings over a long period of time. In their animal and human cadaver study, Barberio et al. used both endoscopic tattooing with ICG and marking with the fluorescent OSC. In assessing visibility of NIR markings during MIS surgery, detection rate of ICG tattoos was only 33–50% 4–6 days after endoscopy. 10–11 days after endoscopic tattooing the detection rate of ICG tattoos decreased to 0%. In contrast, all markings with the fluorescent OSC were visible even 10 days after endoscopic marking [30]. However, fluorescent coating of OSC is time-consuming, which limits their usability in the clinical setting.

Another approach is fluorescence-enhanced visualization of colorectal carcinomas with specific fluorescence probes that can target tumor-specific biomarkers [32]. In this way, only tumor tissue should be selectively marked for a visual distinction between tumor tissue and non-tumor tissue. However, there remain many challenges and many questions that need to be answered to further optimize the use of tumor antigen targeted NIR marking including the ideal probe selection and enhancing visibility [32].

The results of our study indicate further potential applications of this new method of NIR fluorescence tattooing. In our study, endoscopic tattooing with the NIR fluorescent marker AFS81x also resulted in labeling of the locoregional lymph nodes by macrophages that phagocytosed the fluorescent marker and migrated in the locoregional lymph nodes. Possible areas of application such as the identification of sentinel lymph nodes or the identification of tumor draining lymph nodes are conceivable. The new method of NIR fluorescent tattooing could also be used in other organs to mark tumor locations reliably and accurately for surgery. Conceivable applications include gastric and esophageal surgery as well as lung surgery.

Three limitations must be considered with the new method of NIR fluorescent tattooing. The efficacy of our new method of NIR fluorescent tattooing has not yet been shown on patients in clinical practice. In a currently planned clinical trial, the new method will be compared prospectively and randomized with the established method of endoscopic tattooing with SPOT™. Second, the time interval between diagnosis by endoscopy and surgical removal of colorectal cancer in clinical practice is on average 4–6 weeks [14, 33]. The ideal agent should therefore ensure stable marking during this period. This was not revealed in our study because the animal study was terminated after 10 days. Further in vivo studies are needed to demonstrate stability of NIR fluorescence tattooing over a longer period of time. Ex vivo we could show stability of the new fluorescent dye AFS81x over several weeks. Since the configuration of the fluorescence tattoos in our study did not change macroscopically between day 0 and day 10, i.e., did not show any reduction in intensity or significant change in size, it can be assumed that the tattoos remain stable over a longer period of weeks. Third, whether the new fluorescent marker AFS81x can cause an inflammatory response similar to India Ink needs to be investigated in clinical use. However, in histologic examination of the colonic specimens from this animal study, we did not detect any increased inflammatory response to endoscopic fluorescence tattooing. In addition, there were no side effects of endoscopic NIR fluorescent tattooing in our animal study.

In conclusion, our new method of endoscopic NIR fluorescence tattooing using the newly developed NIR fluorescence marker AFS81x addresses almost all requirements of an ideal approach for preoperative marking of colon lesions as (i) it enables a readily and accurately detection during MIS surgery, (ii) it is as practical in clinical use as India ink or SPOT™, (iii) it does not affect the identification of anatomical structures and surgical planes in any way during surgery, and (iv) it enables stable marking over a longer period of at least 10 days. Further studies are needed to demonstrate efficacy of our new approach of NIR fluorescent tattooing in clinical use on patients and to demonstrate stability of marking over a longer period of time.

## Abbreviations

NIR: Near infrared
MIS: Minimally invasive surgery
CT: Computed tomography
MRI: Magnetic resonance imaging
CME: Complete mesocolic excision
ICG: Indocyanine green
